# Genetic variation assessment of stacked-trait transgenic maize via conventional breeding

**DOI:** 10.1186/s12870-019-1956-y

**Published:** 2019-08-07

**Authors:** Xujing Wang, Xin Zhang, Jiangtao Yang, Xiaojing Liu, Yaya Song, Zhixing Wang

**Affiliations:** Biotechnology Research Institute, Chinese Academy of Agricultural Sciences, MARA Key Laboratory on Safety Assessment (Molecular) of Agri-GMO, 12 Zhuangguancun South Street, Beijing, 100081 China

**Keywords:** Stacked trait, Transgenic maize, Genetic stability, Proteomic analysis

## Abstract

**Background:**

The safety assessment and control of stacked transgenic crops is increasingly important due to continuous crop development and is urgently needed in China. The genetic stability of foreign genes and unintended effects are the primary problems encountered in safety assessment. Omics techniques are useful for addressing these problems. The stacked transgenic maize variety 12–5 × IE034, which has insect-resistant and glyphosate-tolerant traits, was developed via a breeding stack using 12–5 and IE034 as parents. Using 12–5 × IE034, its parents (12–5 and IE034), and different maize varieties as materials, we performed proteomic profiling, molecular characterization and a genetic stability analysis.

**Results:**

Our results showed that the copy number of foreign genes in 12–5 × IE034 is identical to that of its parents 12–5 and IE034. Foreign genes can be stably inherited over different generations. Proteomic profiling analysis found no newly expressed proteins in 12–5 × IE034, and the differences in protein expression between 12 and 5 × IE034 and its parents were within the range of variation of conventional maize varieties. The expression levels of key enzymes participating in the shikimic acid pathway which is related to glyphosate tolerance of 12–5 × IE034 were not significantly different from those of its parents or five conventional maize varieties, which indicated that without selective pressure by glyphosate, the introduced EPSPS synthase is not has a pronounced impact on the synthesis of aromatic amino acids in maize.

**Conclusions:**

Stacked-trait development via conventional breeding did not have an impact on the genetic stability of T-DNA, and the impact of stacked breeding on the maize proteome was less significant than that of genotypic differences. The results of this study provide a theoretical basis for the development of a safety assessment approach for stacked-trait transgenic crops in China.

## Background

Stacked genetically modified crops (GMCs) with their improved traits, versatility and low cost, have been well received by many growers and researchers since their inception and are now leading transgenic crop developments. In 2016, stacked GMCs were grown in 14 countries and had a planting area of 75.4 million hectares, accounting for 41% of the global transgenic crop planting area [1]. The rapid application of stacked GMCs has led to concerns over whether the safety of such products differs from that of single-trait products and how the safety of such products will be assessed.

Stacked GMCs can be obtained through cotransformation, retransformation and conventional breeding [2, 3]. Typically, a stacked GMC that is produced by cotransformation and retransformation requires a de novo safety assessment as a new event [4]. However, the requirements for the safety assessment of products obtained using conventional breeding stack strategies are not standardized and differ among countries [5–9]. The main question is whether a breeding stack creates unintended effects and changes that require additional safety assessments. Two primary concerns are 1) whether a breeding stack can increase genomic instability and 2) whether potential interactions between the products of the transgenes in stacked GMCs impact safety [4, 10].

Recently, omics approaches including genomics, transcriptomics, metabolomics and proteomics have provided a valuable platform to analyze the unintended effects of GMCs [11–14]. Proteomic analysis is especially useful to assess unintended effects in GMCs because proteins are responsible for much of plant growth and metabolism [15]. Ren et al. analyzed the impacts of different environmental treatments and genetic modifications on the proteome of *Arabidopsis*. A total of 102 significantly different proteins were detected between 12 transgenic *Arabidopsis* plants featuring different T-DNA insertion sites and wild-type *Arabidopsis*. The impact of cold treatment on the *Arabidopsis* proteome was more significant than that of genetic modification [16]. Proteomics analyses have also been used to test for unintended effects in GMCs including genetically modified (GM) rice [17], oilseed [15], tomato [18], maize [11, 19], wheat [20], pea [21] and tobacco [22]. Most of these studies have found that the percentage of significantly different proteins between transgenic and non-transgenic varieties is very low and that the differences were expected or within the range of natural variation [15, 17]. Gapito-Tenfen et al. [23] reported that protein changes observed in the stacked insecticidal (*cry*) and herbicide tolerance (*epsps*) transgenic maize proteome differed significantly from those of single event lines and a conventional counterpart. Using transcriptomics and metabolomics profile analysis, Wang et al. [24] reported far fewer differences in gene expression and metabolites resulting from the breeding stack than those found among traditional maize varieties. An important issue to address is how altered protein production compares with the range of natural variability after a breeding stack event.

The stacked transgenic maize 12–5 × IE034 which contains the insecticidal *cry* and glyphosate tolerance *G10-epsps* genes was obtained by sexual hybridization of transgenic maize 12–5 and IE034. It has simultaneous resistance to glyphosate and pests. The target genes can be expressed successfully at the RNA and protein level in 12–5 × IE034 [24]. In this study, proteomics was used as a molecular profiling technique to identify potential effects of the breeding stack in GM varieties. We compared the proteomic data of eight maize varieties including 12–5 × IE034, its breeding parents (12–5 and IE034), and five traditional maize varieties, and we evaluated the protein changes due to variety, transformation and the breeding stack. The results indicated that far fewer protein differences resulted from the breeding stack than from transformation and traditional maize varieties. This finding provides a theoretical basis and scientific data for the development of a safety assessment of stacked GMCs in China.

## Results

### Molecular characterization of stacked-trait transgenic maize 12–5 × IE034

**Fig. 1:**
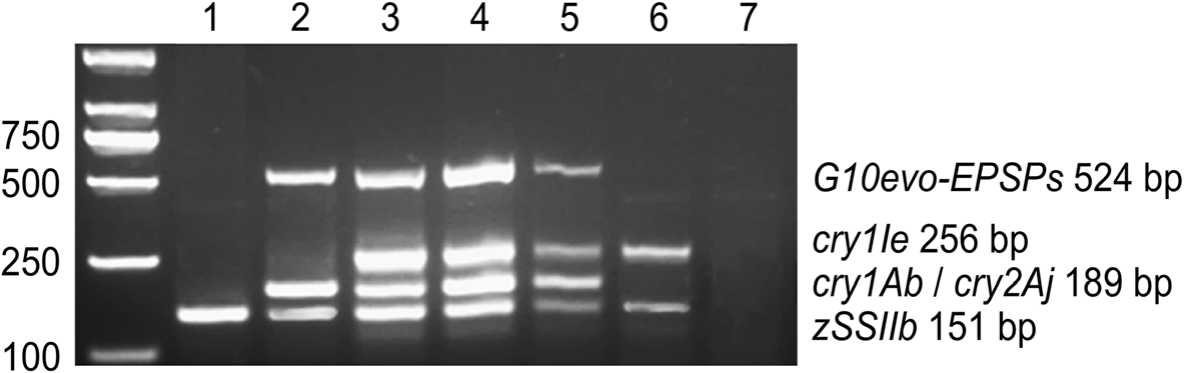
Multiple PCR detection of stacked-trait GM maize 12–5 × IE034 and its self-bred progenies. 1: non-transgenic maize Z58; 2: 12–5; 3: 12–5 × IE034; 4: 12–5 × IE034-F2; 5: 12–5 × IE034-F3; 6: IE034; 7: H_2_O

The multiple PCR detection results showed that all three target genes (*cry1Ie, cry1Ab/cry2Aj* and *G10-EPSPs*) were integrated in stacked-trait GM maize lines 12–5 × IE034, 12–5 × IE034-F2 and 12–5 × IE034-F3, and the sizes of the amplified fragments were consistent with those of the DNA fragments in the corresponding parent maize (Fig. 1).

Micro-droplet digital PCR is an absolute quantification technique for nucleic acid molecules based on the poisson distribution principle and was designed to determine the copy number of foreign DNA quickly and accurately. The primers and probes used in this study showed good specificity and were capable of clearly distinguishing positive and negative micro-droplets. The number of micro-droplets generated in the test was greater than 13,000 which met the poisson distribution criterion. In addition, the relative standard deviation (RSD) value for the number of micro-droplets generated by three wells was smaller than 0.25 which met the EU’s nucleic acid molecule detection requirement. The copy number of the target genes was calculated using a prepared digital PCR system. The copy number of the T-DNA integrated in the genome of the stacked-trait transgenic maize 12–5 × IE034 was 0.47–0.5, wherase that integrated in the parent event was 0.97–1.07 (Table 1). This difference was expected because the heterozygous foreign DNA content in 12–5 × IE034 was theoretically half of that in its transgenic parents 12–5 and IE034. The micro-droplet digital PCR results were consistent with the theoretical value, which indicated that stacked-trait development via conventional breeding is not expected to change the copy number of foreign DNA in the genome.

**Table 1:** Copy numbers of target genes in transgenic maize through micro-droplet digital PCR

Sample	Gene	Total number of micro-droplets	Density (copies/μL)	Copy number
IE034	*Hmga*	14,406 ± 1001	265 ± 18.25	
	*cry1Ie*	15,414 ± 1181	250 ± 40.67	0.94
12–5 × IE034	*Hmga*	16,071 ± 810	274 ± 5.57	
	*cry1Ie*	18,776 ± 332	128 ± 5.13	0.47
	*cry1Ab*	18,484 ± 289	135 ± 3.79	0.49
	*G10-EPSPs*	18,439 ± 730	136 ± 3.61	0.5
12–5	*Hmga*	17,321 ± 510	264 ± 15.40	
	*cry1Ab*	16,930 ± 1741	268 ± 34.95	1.02
	*G10-EPSPs*	17,573 ± 322	282 ± 18.82	1.07

### Determination of the expression levels of foreign genes in stacked-trait transgenic maize 12–5 × IE034

The results of real-time PCR indicated that the expression of the target genes in 12–5 × IE034 was lower than that in the parents 12–5 and IE034. The expression levels of *cry1Ab* and *G10-EPSPs* in 12–5 were nearly twice those of 12–5 × IE034. The expression level of *cry1Ie* in IE034 was 2.5-fold that of 12–5 × IE034.

**Fig. 2:**
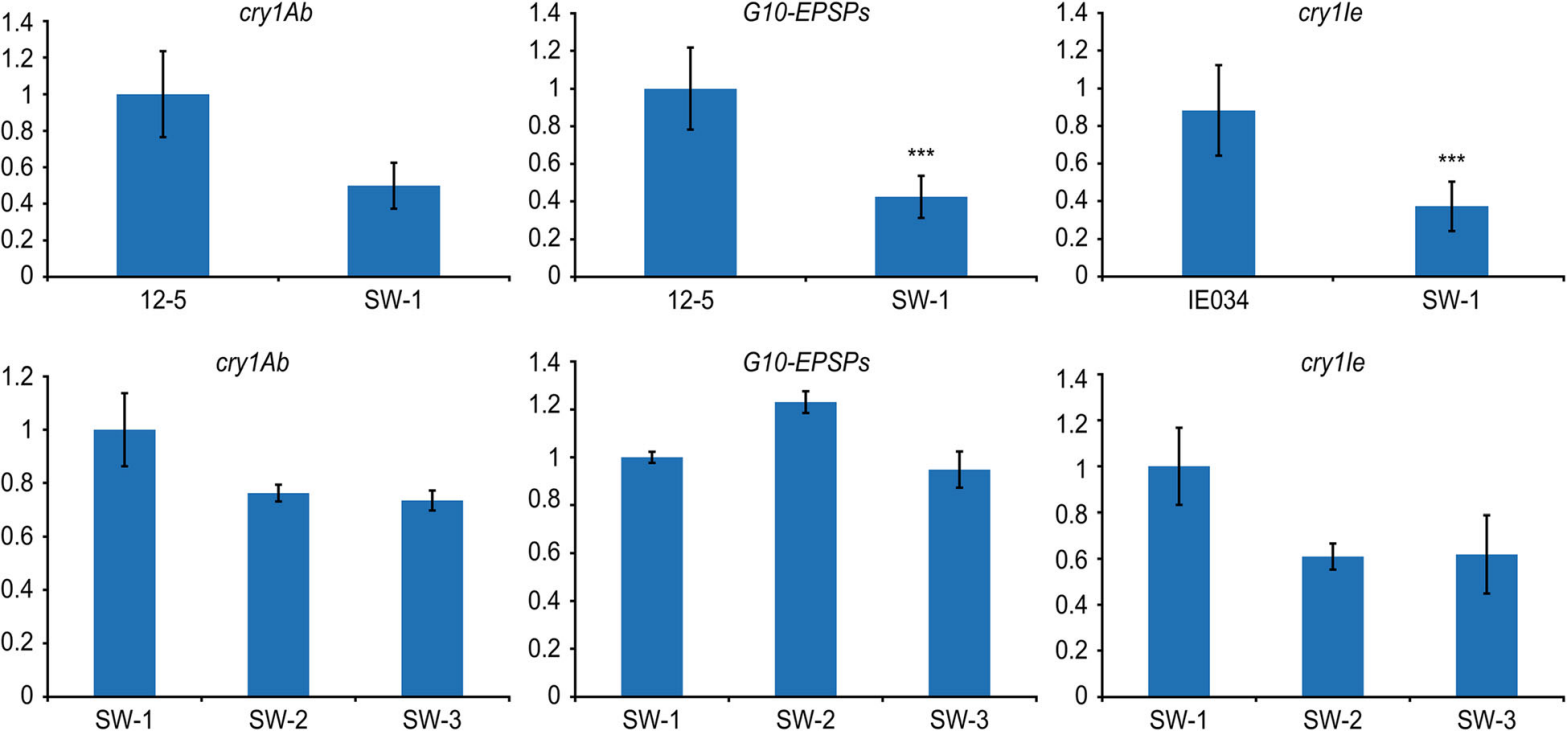
Transgene transcript normalized relative expression levels measured by qRT-PCR. The *cry1Ab*, *G10-EPSPs* and *cry1Ie* transgenes were quantified from stacked transgenic maize and single transgenic maize events grown under the same conditions. Data are means of three pools, each derived from five different plants. SW-1 samples are from stacked transgenic maize 12–5 × IE034, 12–5 samples are from transgenic maize 12–5, IE034 samples are from transgenic maize IE034, SW-2 samples are from transgenic maize 12–5 × IE034-F2, and SW-3 samples are from transgenic maize 12–5 × IE034-F3. Bars indicate standard deviations, and statistically significant values (*P* < 0.05) are indicated by ‘**’

Regarding the different generations of 12–5 × IE034, the expression levels of *cry1Ie* and *cry1Ab* in 12–5 × IE034 and 12–5 × IE034-F2 were higher than those in 12–5 × IE034-F3. In addition, the expression level of *G10-EPSPs* in 12–5 × IE034-F3 was higher than the corresponding levels in 12–5 × IE034 and 12–5 × IE034-F2. However, these expression level changes were not significantly different (using a significance level of 0.05) based on an analysis using SPSS software (Fig. 2).

### Inter-maize variety proteome analysis

**Fig. 3:**
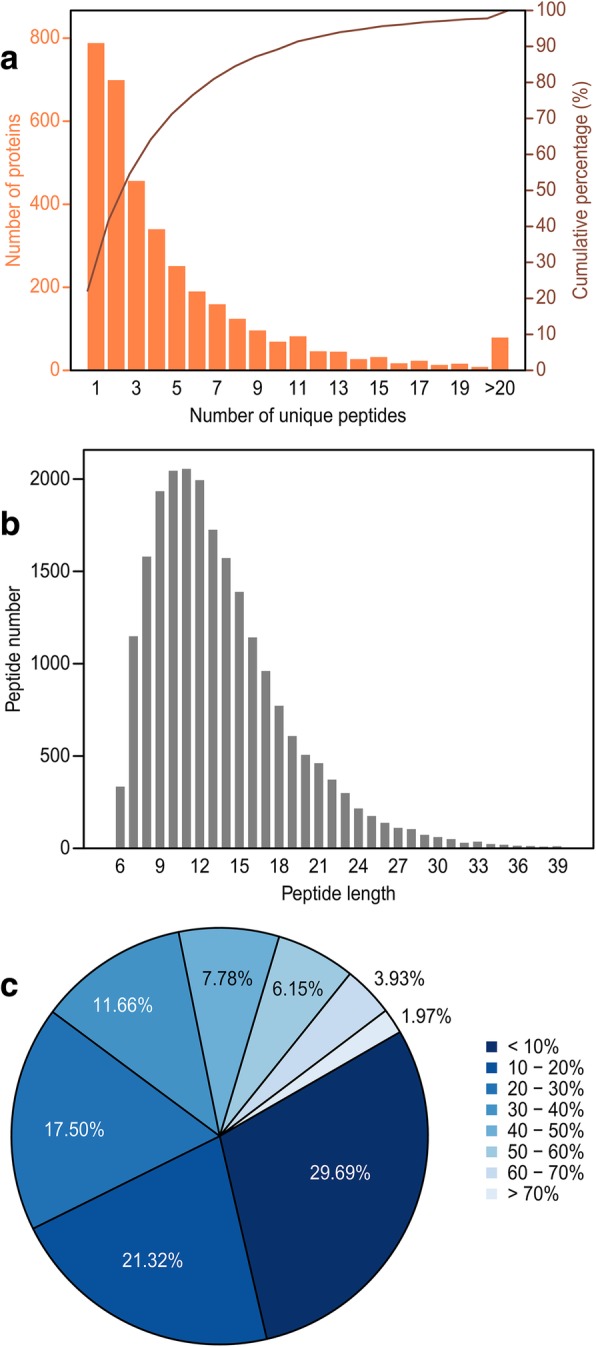
Quality analysis of proteomic profiling. a: Distribution of unique peptides. The abscissa presents the unique peptide numbers of the identified proteins. The vertical ordinate at left denotes the protein number corresponding to the abscissa value. The vertical ordinate at right denotes the cumulative protein ratio. Proteins containing at least 2 peptides represented 77.87% of the identified proteins. b: Distribution of peptide lengths. Most peptides had a length of 11. The average peptide length was 13.55 which is within the reasonable range. c: Coverage of identified proteins. Different colors indicate different identification coverage ranges of proteins. The identification coverage of 29.69% of the proteins was between 0 and 10%. The identification coverage of 48.99% of the proteins was higher than 20%. The average protein identification coverage was 23.93%

In this study, mass spectrometry generated 449,238 s-level patterns. The number of identified patterns was 164,031 yielding an identification rate of 36.51%. In addition, 21,837 peptides were identified and the average peptide length was 13.55 amino acids, which was within a reasonable range of peptide lengths. A total of 3560 proteins were identified yielding an average protein identification coverage of 23.93%. In addition, 2772 proteins containing at least two unique peptides were identified, accounting for 77.87% of the total number of identified proteins (Fig. 3).

### Proteomic analysis of genotype effects

**Fig. 4:**
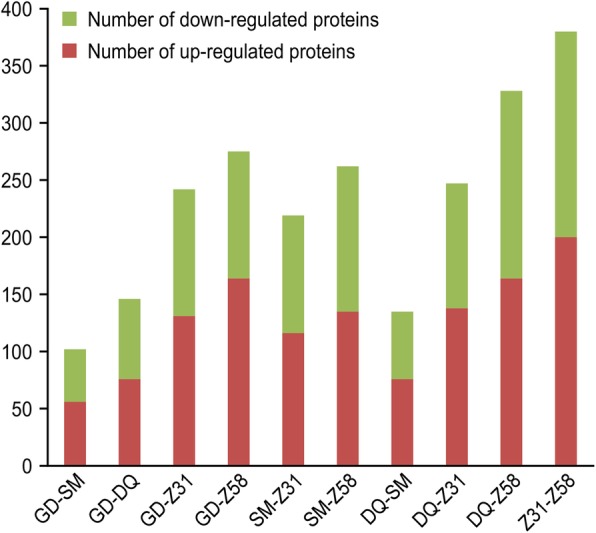
Pairwise comparisons of differentially expressed proteins between traditional maize varities. SM: traditional maize cultivar Sanmingzi; DQ: traditional maize cultivar Daqingke; BM: traditional maize cultivar Baimaya; Z31: traditional maize cultivar Zong31, the recipient of IE034; Z58: traditional maize cultivar Zhengdan58, the recipient of 12–5. The number of differentially expressed proteins in the various traditional maize varieties ranged from 102 to 380. The largest difference was observed between Z31 and Z58

The number of differentially expressed proteins in the various traditional maize varieties ranged from 102 to 380 (Fig. 4). The highest number of differentially expressed proteins (380) was observed between Z58 and Z31 which are the two parental varieties used to establish 12–5 and IE034, respectively. This result futher demonstrated that the genetic distances of the two parental varieties of the transgenic maize are long. Gene Ontology (GO) analysis showed that the differentially expressed proteins were primarily associated with the following terms: metabolic process and cellular process in the biological process category, cell and cell part in the cellular component category, and binding and antioxidant in the catalytic activity category. Kyoto Encyclopedia of Genes and Genomes (KEGG) pathway analysis showed that the differentially expressed proteins were primarily related to metabolic pathways, biosynthesis of secondary metabolites, ribosomes, and microbial metabolism in diverse environments.

### Proteomic analysis of the transgene effects

**Fig. 5:**
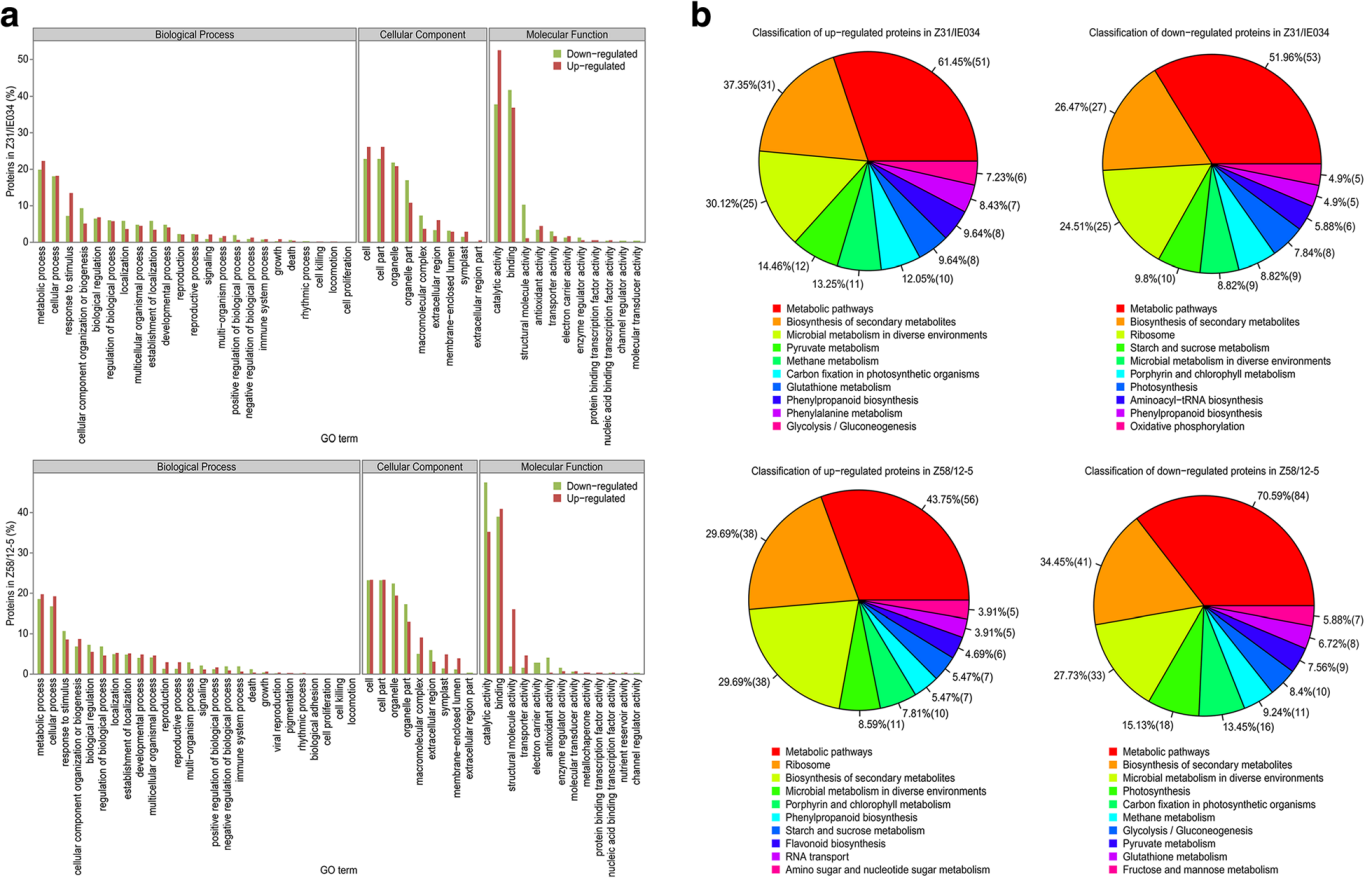
GO and KEGG analysis of differentially expressed proteins between transgenic maize and its recipients. **a** GO analysis. Z31/IE034, the GO analysis results of differentially expressed proteins between transgenic maize IE034 and its recipient Z31. Z58/12–5, the GO analysis results of differentially expressed proteins between transgenic maize 12–5 and its recipient Z58. The red column presents the functional classification ratios of up-regulated proteins under the categories biological progress, cellular component and molecular function. The green column presents the functional classification ratios of down-regulated proteins under the categories biological progress, cellular component and molecular function. **b** KEGG analysis results of differentially expressed proteins from Z31/IE034 and Z58/12–5. The percentages of the top ten pathways related to the differentially expressed proteins are shown. The number of proteins in each pathway is shown in parentheses

A total of 264 and 397 differentially expressed proteins were found between IE034 and its recipient Z31, and between 12 and 5 and its recipient Z58, respectively. GO analysis showed that the proteins were associated with the following terms: metabolic process, cellular process, cell, cell part, organelle, binding and catalytic activity (Fig. 5a). KEGG analysis indicated that these proteins were primarily related to metabolic pathways and biosynthesis of secondary metabolites (Fig. 5b).

**Fig. 6:**
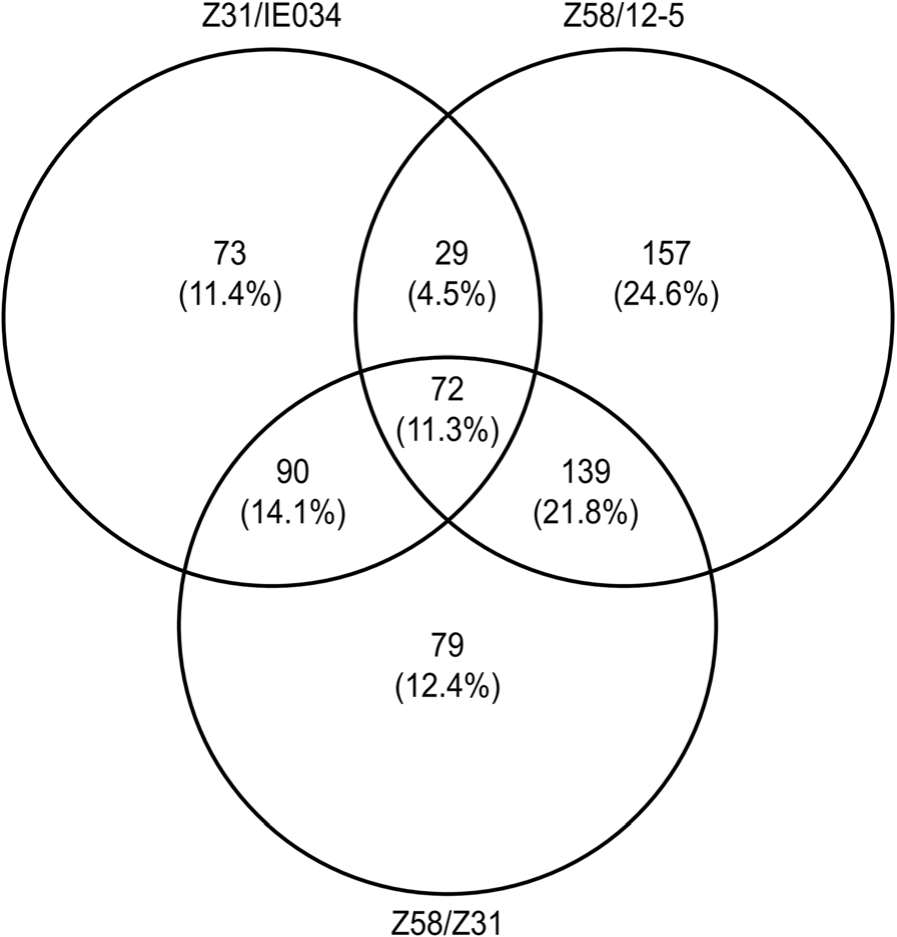
Venn diagram of differentially expressed proteins due to transgenic effects. Z31/IE034, proteomic comparison of the transgenic maize (IE034) and its recipients (nontransgenic maize Zong31) for detecting transgenic effects. Z58/12–5, proteomic comparison of the transgenic maize (12–5) and its recipients (nontransgenic maize Zhengdan58) for detecting transgenic effects. Z58/Z31, proteomic comparison of the traditional maize cultivar Zhengdan58 and Zong31 for detecting differences between recipients

Correlation analysis of differentially expressed proteins among IE034, 12–5 and the recipients Z31 and Z58 through pairwise comparisons showed 72 common differentially expressed proteins in the comparison groups of IEO34/Z31, Z58/Z31 and 12–5/Z58. In the pairwise comparison of groups, 73, 79 and 157 proteins were exclusively differentially expressed in Z31/IE034, Z31/Z58, and Z58/12–5, respectively (Fig. 6). These differentially expressed proteins were neither toxins nor allergens, and almost all were associated with the terms metabolic process, cellular process, cell, cell part, organelle, binding and catalytic activity.

### Proteomic analysis of stack breeding effects

**Fig. 7:**
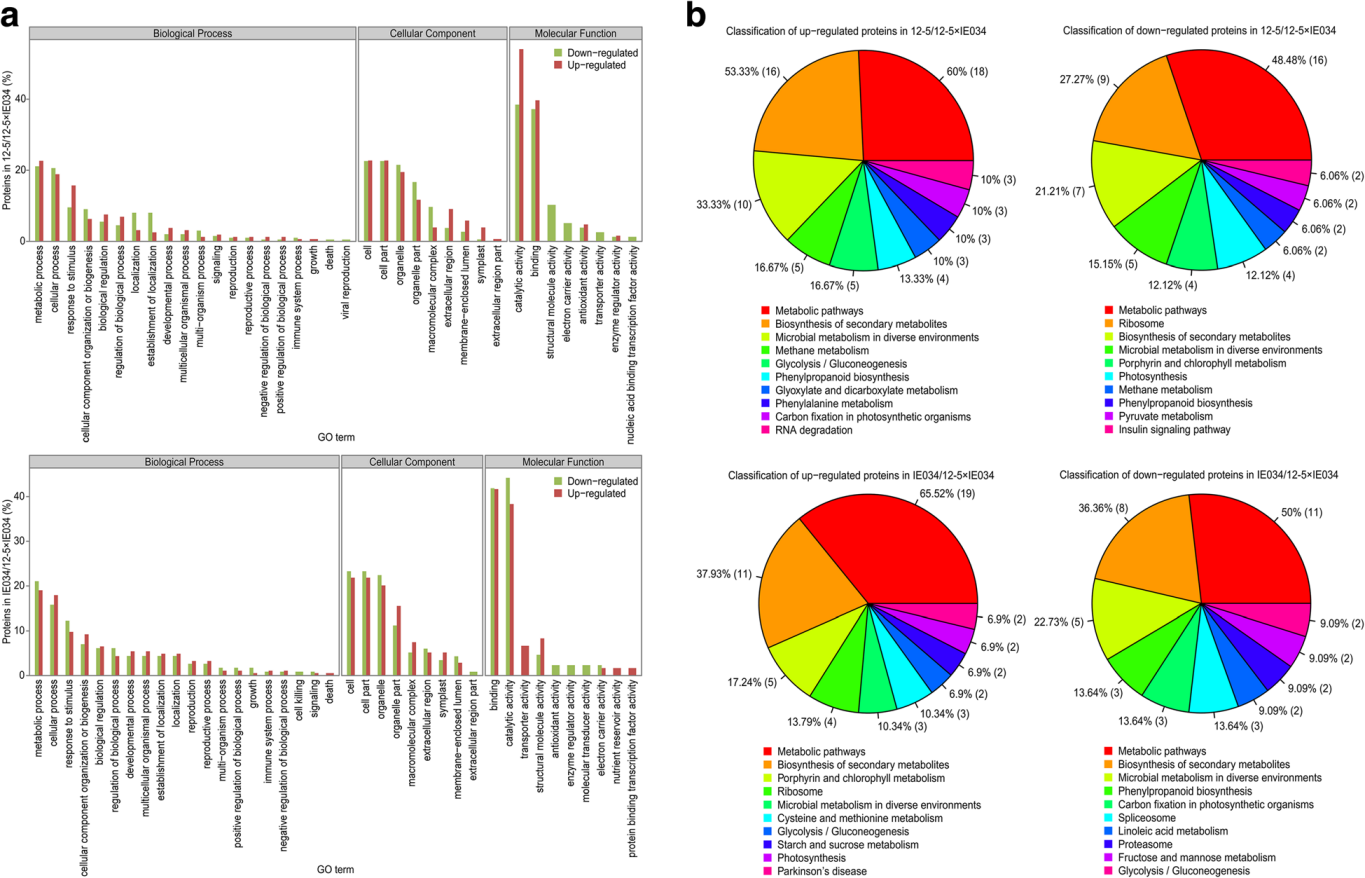
GO and KEGG analysis of differentially expressed proteins between 12 and 5 × IE034 and its parents. **a** GO analysis of differentially expressed proteins between 12 and 5 × IE034 and its parents. 12–5/12–5 × IE034, the GO analysis results of differentially expressed proteins between transgenic maize 12–5 × IE034 and its parent 12–5. IE034/12–5 × IE034, the GO analysis results of differentially expressed proteins between transgenic maize 12–5 × IE034 and its parent 12–5. The red column presents the functional classification ratios of upregulated proteins under the categories biological progress, cellular component and molecular function. The green column presents the functional classification ratios of downregulated proteins under the categories biological progress, cellular component and molecular function. **b** KEGG analysis results of differentially expressed proteins between 12 and 5 × IE034 and its parents. The percentages of the top ten pathways related to the differentially expressed proteins are shown. The number of proteins in each pathway is shown in parentheses

Relative to its parents 12–5 and IE034, 12–5 × IE034 contained 72 and 87 differentially expressed proteins, respectively. These differentially expressed proteins were primarily associated with the following GO terms: metabolic process, cellular process and response to stimulus in the biological process category, cell, cell part and organelle in the cellular component category, and catalytic activity and binding in the molecular function category (Fig. 7a). In the KEGG analysis, the differentially expressed proteins were primarily related to metabolic pathways, biosynthesis of secondary metabolites, ribosomes and microbial metabolism in diverse environments (Fig. 7b). These results indicated that the functions of the differentially expressed proteins between 12 and 5 × IE034 and its parents are similar to those of proteins differentially expressed among other maize varieties.

**Fig. 8:**
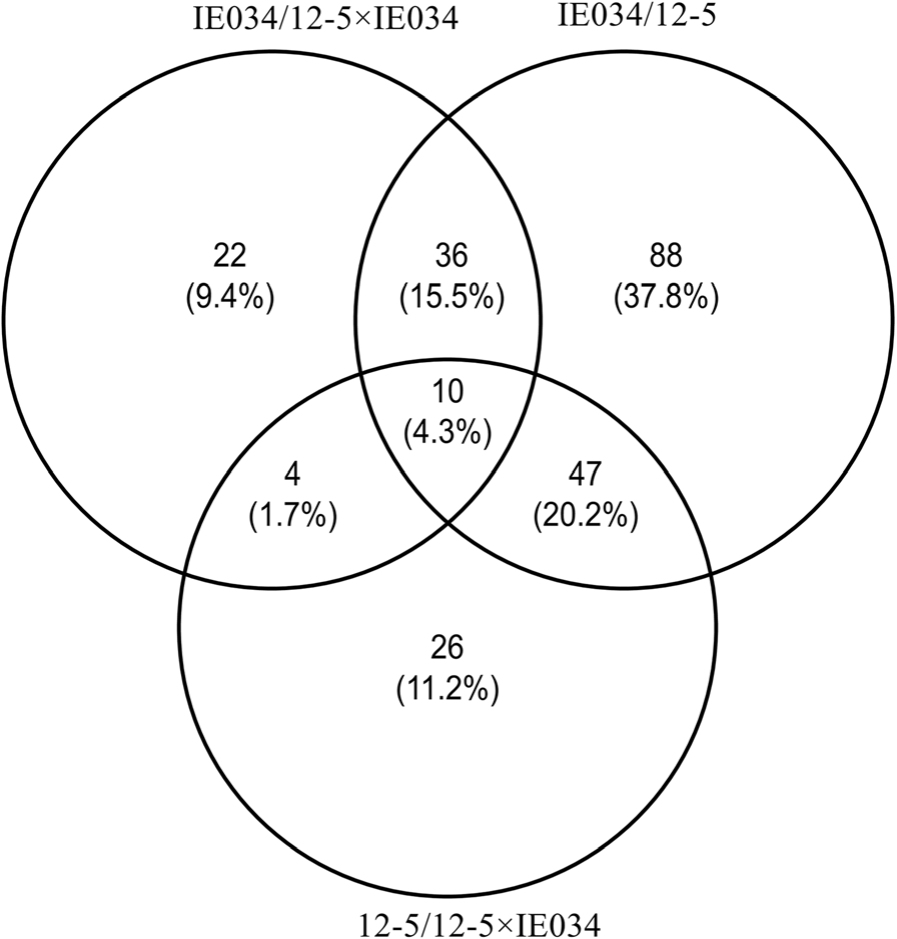
Venn diagram of differentially expressed proteins due to breeding stack. IE034/12–5 × IE034, proteomic comparison of the stacked transgenic maize 12–5 × IE034 and its parent IE034 for detecting stack effects. 12–5/12–5 × IE034, proteomic comparison of the stacked transgenic maize 12–5 × IE034 and its parent 12–5 for detecting stack effects. IE034/12–5, proteomic comparison of the transgenic maize 12–5 and IE034 for detecting differences between the breeding parents

A correlation analysis of differentially expressed proteins among IE034, 12–5 and 12–5 × IE034 through pairwise comparisons showed 10 common differentially expressed proteins in the comparison groups of IEO34/12–5 × IE034, 12–5/IE034 and 12–5/12–5 × IE034. In the pairwise comparison of 12–5 × IE034/IE034 and 12–5 × IE034/12–5, 22 and 26 proteins were exclusively differentially expressed, respectively. These differentially expressed proteins were neither toxins nor allergens, and almost all included the following GO terms: metabolic process, cellular process, cell, cell part, organelle, binding and catalytic activity (Fig. 8). In addition, all of these proteins were identified in and differentially expressed among various traditional maize varieties (Table 2). No unintended new proteins were found in the 12–5 × IE034 proteome; i.e., only the target foreign proteins were found.

**Table 2:** Exclusively differentially expressed proteins in IE034 /12–5 × IE034 and 12–5/12–5 × IE034

Accession	Name	IE034/SW-1	PVal IE034/SW-1	12–5/ SW-1	PVal 12–5/ SW-1	Z31/Z58	PVal Z31/Z58	SM/Z58	PVal SM/Z58	DQ/Z58	PVal DQ/Z58	BM/Z58	PVal BM/Z58	SM/Z31	PVal SM/Z31	DQ/Z31	PVal DQ/Z31	BM/Z31	PVal BM/Z31	DQ/SM	PVal DQ/SM	BM/SM	PVal BM/SM	BM/DQ	PVal BM/DQ
A0A1D6GQ42_MAIZE	Uncharacterized protein	1.43	0.31	2.31	0.04	0.46	0.08	0.54	0.04	0.44	0.03	0.50	0.03	1.17	0.73	0.93	0.55	1.05	0.54	0.81	0.79	0.90	0.78	1.14	0.99
A0A1D6PUL3_MAIZE	Aconitate hydratase	1.45	0.20	1.71	0.04	1.82	0.01	0.91	0.99	0.91	0.52	1.03	0.51	0.51	0.01	0.48	0.00	0.58	0.00	1.00	0.46	1.13	0.38	1.15	0.89
K7U9U9_MAIZE	Ferredoxin-nitrite reductase	0.86	0.33	0.65	0.01	0.84	0.37	2.36	0.01	1.50	0.40	2.94	0.00	2.73	0.00	1.82	0.09	3.47	0.00	0.62	0.08	1.26	0.22	2.03	0.00
B6SHD8_MAIZE	Non-cyanogenic ß-glucosidase	0.86	0.38	0.60	0.01	0.98	0.57	1.27	0.14	0.49	0.10	0.29	0.02	1.27	0.33	0.48	0.03	0.28	0.01	0.38	0.01	0.23	0.00	0.58	0.40
tr|Q5EUE1|Q5EUE1_MAIZE	Protein disulfide-isomerase	1.31	0.13	2.01	0.00	1.10	0.51	1.16	0.48	1.06	0.64	0.82	0.28	1.05	0.96	0.95	0.84	0.74	0.10	0.91	0.81	0.70	0.09	0.78	0.13
tr|C4JAX7|C4JAX7_MAIZE	UDP-sulfoquinovose synthase	1.25	0.42	1.85	0.00	1.64	0.02	0.63	0.14	0.94	0.68	1.25	0.72	0.38	0.00	0.59	0.01	0.74	0.04	1.56	0.28	1.94	0.07	1.29	0.44
tr|C4J4E4|C4J4E4_MAIZE	Uncharacterized protein	2.75	0.07	3.19	0.01	2.00	0.00	0.94	0.91	0.91	0.96	0.80	0.52	0.47	0.00	0.47	0.00	0.40	0.00	0.98	0.87	0.85	0.60	0.86	0.49
tr|B6SRJ5|B6SRJ5_MAIZE	Bifunctional 3-phosphoadenosine 5-phosphosulfate synthetase 2	0.69	0.00	0.63	0.03	0.93	0.86	1.54	0.24	1.84	0.07	2.01	0.01	1.63	0.19	1.98	0.05	2.11	0.01	1.20	0.47	1.33	0.06	1.11	0.23
tr|B4FNZ1|B4FNZ1_MAIZE	Sucrose-phosphatase 1	1.41	0.35	1.71	0.01	1.12	0.48	1.01	0.78	0.74	0.39	0.30	0.03	0.90	0.66	0.69	0.12	0.28	0.01	0.75	0.26	0.30	0.01	0.39	0.09
tr|K7TFW8|K7TFW8_MAIZE	Uncharacterized protein	0.86	0.42	0.60	0.02	0.44	0.00	0.54	0.01	0.78	0.24	0.63	0.06	1.26	0.15	1.79	0.01	1.46	0.03	1.47	0.11	1.18	0.39	0.79	0.42
tr|A0A1D6EFP8|A0A1D6EFP8_MAIZE	Uncharacterized protein	0.82	0.15	0.58	0.01	0.38	0.01	0.93	0.14	0.77	0.34	0.34	0.00	2.42	0.15	2.03	0.03	0.94	0.19	0.82	0.60	0.36	0.01	0.44	0.00
tr|Q8W0Q2|Q8W0Q2_MAIZE	Putative aldehyde dehydrogenase MIS1	1.13	0.77	1.60	0.03	1.51	0.15	1.16	0.61	1.42	0.17	1.06	0.78	0.76	0.19	0.93	0.72	0.69	0.03	1.24	0.29	0.90	0.40	0.73	0.07
tr|B4F7Z4|B4F7Z4_MAIZE	Uncharacterized protein	1.47	0.26	2.01	0.00	9.04	0.00	0.54	0.96	2.75	0.03	2.36	0.19	0.06	0.00	0.26	0.00	0.23	0.00	5.06	0.00	4.09	0.04	0.91	0.50
tr|K7V8R6|K7V8R6_MAIZE	Peptidyl-prolyl cis-trans isomerase	0.70	0.13	0.31	0.02	0.55	0.31	0.95	0.96	0.95	0.67	0.19	0.10	1.74	0.23	1.72	0.12	0.33	0.09	1.00	0.65	0.20	0.05	0.20	0.02
tr|B6THU9|B6THU9_MAIZE	Peroxidase	0.90	0.44	0.54	0.04	0.95	0.63	1.32	0.24	1.77	0.02	0.86	0.48	1.36	0.12	1.80	0.01	0.86	0.80	1.37	0.17	0.66	0.08	0.49	0.01
tr|C0PNM2|C0PNM2_MAIZE	Uncharacterized protein	0.73	0.61	0.59	0.04	1.03	0.83	0.81	0.90	0.53	0.59	1.94	0.09	0.72	0.74	0.58	0.29	1.84	0.15	0.78	0.67	2.63	0.09	3.44	0.05
tr|A0A1D6N3I1|A0A1D6N3I1_MAIZE	Uncharacterized protein	0.82	0.40	0.53	0.03	4.29	0.00	2.94	0.10	1.47	0.49	3.28	0.03	0.67	0.07	0.35	0.01	0.67	0.14	0.51	0.22	1.03	0.74	1.96	0.17
tr|B6TGK1|B6TGK1_MAIZE	Ribonuclease 2	1.15	0.65	2.44	0.04	2.03	0.06	1.06	0.55	1.01	0.96	1.25	0.36	0.49	0.14	0.47	0.06	0.59	0.23	0.97	0.57	1.15	0.72	1.27	0.33
tr|B4FR21|B4FR21_MAIZE	ATP-dependent RNA helicase dhh1	0.88	0.43	0.64	0.05	0.73	0.11	0.74	0.23	0.51	0.02	0.66	0.05	1.01	0.61	0.68	0.30	0.90	0.60	0.67	0.14	0.88	0.32	1.31	0.59
sp|Q10717|CYSP2_MAIZE	Cysteine proteinase 2	0.50	0.31	2.88	0.02	5.11	0.07	4.33	0.16	2.58	0.26	2.49	0.32	0.88	0.51	0.47	0.04	0.52	0.24	0.56	0.35	0.63	0.60	1.09	0.63
tr|A0A1D6P4W5|A0A1D6P4W5_MAIZE	Uncharacterized protein	0.55	0.07	0.52	0.02	0.88	0.55	0.95	0.55	1.14	0.71	0.84	0.54	1.06	0.75	1.28	0.93	0.95	0.68	1.16	0.82	0.90	0.65	0.74	0.70
tr|B4FU15|B4FU15_MAIZE	Uncharacterized protein	0.79	0.53	0.26	0.04	1.66	0.30	2.17	0.15	3.10	0.04	2.54	0.07	1.32	0.60	1.89	0.14	1.53	0.28	1.43	0.27	1.16	0.54	0.81	0.58
tr|C0P456|C0P456_MAIZE	Uncharacterized protein	0.79	0.42	0.60	0.04	0.73	0.24	0.88	0.34	0.80	0.29	0.69	0.10	1.20	0.80	1.08	0.88	0.92	0.57	0.91	0.91	0.79	0.41	0.87	0.47
sp|Q9FQA3|GST23_MAIZE	Glutathione transferase GST 23	1.18	0.00	1.84	0.00	5.60	0.02	1.61	0.46	1.89	0.19	1.43	0.41	0.27	0.02	0.32	0.03	0.24	0.01	1.17	0.41	0.86	0.19	0.75	0.10
sp|Q43298|CH62_MAIZE	Chaperonin CPN60–2, mitochondrial	1.50	0.00	1.74	0.00	0.92	0.63	0.87	0.03	1.09	0.17	1.02	0.51	0.92	0.04	1.19	0.12	1.10	0.30	1.26	0.00	1.17	0.00	0.94	0.32
tr|B6TXC2|B6TXC2_MAIZE	Isopenicillin N epimerase	1.24	0.04	0.39	0.04	0.21	0.24	0.86	0.70	0.65	0.53	0.66	0.41	3.94	0.29	2.99	0.35	2.96	0.44	0.75	0.76	0.77	0.55	1.02	0.73
tr|A0A1D6DSB5|A0A1D6DSB5_MAIZE	Uncharacterized protein	1.89	0.00	1.38	0.01	0.30	0.00	0.29	0.00	0.41	0.00	0.29	0.00	0.93	0.62	1.38	0.07	0.95	0.66	1.50	0.03	1.02	0.87	0.69	0.02
tr|B6SXW8|B6SXW8_MAIZE	RuBisCO large subunit-binding protein subunit alpha	1.54	0.02	1.45	0.00	0.74	0.16	0.28	0.00	0.62	0.04	0.33	0.00	0.38	0.00	0.85	0.48	0.44	0.00	2.19	0.00	1.18	0.35	0.53	0.00
tr|B6TNR8|B6TNR8_MAIZE	40S ribosomal protein S2	3.10	0.03	0.98	0.44	0.25	0.01	0.46	0.06	0.28	0.01	0.37	0.02	1.89	0.14	1.12	0.98	1.49	0.48	0.57	0.15	0.80	0.38	1.39	0.49
tr|C0PEH3|C0PEH3_MAIZE	Uncharacterized protein	1.51	0.01	1.22	0.23	1.13	0.68	0.89	0.29	0.84	0.40	1.10	0.43	0.78	0.15	0.74	0.22	0.95	0.70	0.94	0.82	1.22	0.08	1.32	0.12
tr|K7U7K6|K7U7K6_MAIZE	Uncharacterized protein	0.54	0.02	0.90	0.50	1.53	0.28	1.43	0.19	1.82	0.13	1.43	0.24	0.93	0.42	1.21	0.25	0.92	0.48	1.32	0.08	1.00	0.83	0.79	0.20
tr|K7UXK5|K7UXK5_MAIZE	Putative alcohol dehydrogenase superfamily protein	1.79	0.03	1.00	0.29	1.38	0.24	0.76	0.11	1.08	0.52	1.21	0.62	0.54	0.01	0.77	0.57	0.86	0.48	1.41	0.03	1.58	0.06	1.12	0.89
tr|J9SKH3|J9SKH3_MAIZE	Divinyl reductase	1.77	0.04	1.28	0.47	0.41	0.00	0.94	0.62	0.74	0.07	1.09	0.66	2.29	0.01	1.77	0.11	2.61	0.00	0.79	0.17	1.14	0.36	1.47	0.04
tr|B6U3A0|B6U3A0_MAIZE	Glycine-rich RNA-binding protein 7	0.51	0.00	0.72	0.16	1.05	0.44	1.39	0.23	1.91	0.01	1.56	0.08	1.29	0.66	1.72	0.09	1.43	0.16	1.37	0.19	1.13	0.32	0.82	0.73
tr|Q38JE7|Q38JE7_MAIZE	Temperature-induced lipocalin-1	1.51	0.03	1.03	0.29	5.60	0.00	1.96	0.08	3.50	0.04	2.13	0.19	0.36	0.01	0.65	0.68	0.39	0.20	1.77	0.22	1.05	0.68	0.57	0.11
tr|B6TBW2|B6TBW2_MAIZE	ATP synthase delta chain	2.01	0.04	1.26	0.36	1.13	0.93	0.64	0.15	0.74	0.09	0.48	0.02	0.58	0.13	0.65	0.08	0.42	0.02	1.14	0.74	0.74	0.30	0.65	0.36
tr|Q6QP36|Q6QP36_MAIZE	26S proteasome regulatory complex ATPase RPT3	0.60	0.04	0.90	0.44	0.63	0.05	0.93	0.77	1.13	0.66	0.97	0.69	1.45	0.08	1.79	0.03	1.51	0.10	1.22	0.47	1.05	0.91	0.86	0.41
tr|B7ZYH5|B7ZYH5_MAIZE	Putative O-glycosyl hydrolase superfamily protein	0.44	0.05	0.58	0.15	0.66	0.65	1.56	0.13	1.39	0.20	1.39	0.54	2.31	0.19	2.05	0.26	2.01	0.53	0.87	0.73	0.86	0.33	0.98	0.51
tr|B6SQG1|B6SQG1_MAIZE	Nudix hydrolase 13	0.44	0.03	0.61	0.34	1.80	0.21	0.72	0.74	1.39	0.78	1.91	0.21	0.46	0.55	0.72	0.73	1.04	0.62	1.74	0.51	2.56	0.43	1.49	0.55
tr|D2DWP1|D2DWP1_MAIZE	3-ketoacyl-CoA synthase	2.96	0.02	0.96	0.74	0.11	0.08	0.08	0.02	0.18	0.00	0.15	0.00	0.83	0.60	2.07	0.41	1.57	0.44	2.56	0.35	2.33	0.36	0.86	0.58
tr|B8A0D8|B8A0D8_MAIZE	Lipoxygenase	0.39	0.04	0.38	0.10	0.34	0.00	0.15	0.00	0.26	0.00	0.08	0.00	0.40	0.19	0.75	0.41	0.19	0.14	1.82	0.73	0.48	0.30	0.23	0.21
tr|A0A1D6ICG6|A0A1D6ICG6_MAIZE	Glycosyltransferase	0.52	0.05	0.96	0.98	2.49	0.48	3.70	0.12	3.13	0.29	1.63	0.89	1.29	0.89	1.20	0.86	0.83	0.35	0.90	0.53	0.50	0.16	0.56	0.24
tr|B4FSV7|B4FSV7_MAIZE	Serine/threonine-protein phosphatase	0.59	0.04	0.82	0.41	0.74	0.23	1.10	0.96	0.91	0.73	0.95	0.59	1.45	0.25	1.22	0.36	1.25	0.47	0.84	0.77	0.86	0.62	1.04	0.84
tr|A0A1D6GU11|A0A1D6GU11_MAIZE	Uncharacterized protein	0.56	0.02	0.76	0.21	1.22	0.32	1.07	0.92	1.22	0.30	0.57	0.02	0.86	0.35	0.99	0.96	0.45	0.02	1.15	0.33	0.52	0.02	0.47	0.02
tr|B6TF18|B6TF18_MAIZE	Membrane steroid-binding protein 1	4.61	0.02	2.54	0.10	0.56	0.13	0.55	0.17	0.65	0.32	0.77	0.38	0.96	0.87	1.14	0.50	1.31	0.42	1.17	0.60	1.38	0.51	1.18	0.88
tr|B6SHW0|B6SHW0_MAIZE	60S ribosomal protein L6	1.94	0.03	0.83	0.03	0.47	0.07	1.28	0.59	0.79	0.21	0.96	0.40	2.56	0.03	1.66	0.04	1.92	0.03	0.62	0.27	0.74	0.64	1.21	0.35
tr|C0PKF9|C0PKF9_MAIZE	Cytokinin riboside 5′-monophosphate phosphoribohydrolase	0.44	0.05	0.65	0.21	0.13	0.08	0.51	0.32	0.13	0.09	1.03	0.76	3.60	0.08	1.19	0.04	7.80	0.08	0.27	0.13	1.92	0.35	6.49	0.18

Note: SW-1 presents 12–5 × IE034

### Effect of stacked breeding on the shikimic acid pathway

GO and KEGG analysis of the identified proteins resulted in 70 proteins annotated to the biosynthesis pathways of lysine, tyrosine and phenylalanine, including such key enzymes as chorismic acid synthetase, EPSP synthase and shikimate kinase. Analysis of the protein expression levels indicated that the expression levels of the key enzymes participating in the shikimic acid pathway were not significantly different between the stacked-trait GM maize 12–5 × IE034 and each of its parents 12–5 and IE034, or any of the five conventional maize varieties. This result indicated that without the presence of selective pressure by glyphosate, integration of the gene encoding the EPSPS protein is not expected to produce a marked impact on the shikimic acid pathway or on the synthesis of plant aromatic amino acids (Table 3).

**Table 3:** Protein expression analysis of enzymes related to aromatic amino acid synthesis in different maize varieties

Accession	Name	IE034/ SW-1	12_5/ SW-1	Z58/ SW-1	Z31/ SW-1	SM/ SW-1	BM/ SW-1	Z31/Z58	BM/Z58	BM/Z31
tr|B6TNV8	Shikimate kinase family protein	–	–	–	–	–	–	–	–	–
tr|B4FLA2	Chorismate synthase	–	–	–	–	–	–	–	–	–
tr|B4FTP6	Chorismate synthase	–	–	–	–	–	–	–	–	–
tr|A0A1D6NVZ6	3-phosphoshikimate 1-carboxyvinyltransferase	–	–	–	–	–	–	–	–	–
tr|Q45KJ2	Anthranilate synthase alpha subunit	–	–	–	–	–	–	–	–	–
tr|B6UAK5	Phospho-2-dehydro-3-deoxyheptonate aldolase	–	–	–	–	–	–	–	–	–
tr|B4FUH2	Aspartate aminotransferase	–	–	–	–	–	–	–	–	down
tr|B6T9J4	Aspartate aminotransferase	–	–	–	–	–	–	–	–	–
tr|B4F9G1	Aspartate aminotransferase	–	–	–	down	–	–	–	–	up
tr|B6U1Y5	Transaminase/transferase, transferring nitrogenous groups	–	–	–	–	–	–	up	–	–
tr|B4FAK2	Shikimate dehydrogenase	–	–	–	–	–	–	–	–	down
tr|B6SJH0	3-dehydroquinate synthase	–	–	–	–	–	–	–	–	–
tr|B6TI69	Indole-3-glycerol phosphate lyase	–	–	–	–	–	down	–	down	–
tr|B6TK79	Aspartate aminotransferase	–	–	–	–	–	–	–	–	–
tr|B6TJE4	Anthranilate synthase component II	–	–	–	–	–	–	–	–	–
tr|K7WHC8	Blue fluorescent 1	–	–	–	–	–	–	–	–	–
tr|K7TR93	Tryptophan synthase	–	–	–	–	–	–	down	–	up
tr|B6TUB8	Phospho-2-dehydro-3-deoxyheptonate aldolase	–	–	–	–	–	–	–	–	–
tr|B6UCV5	Shikimate biosynthesis protein aroDE	–	–	–	–	–	–	–	–	–
tr|K7 V589	N-(5′-phosphoribosyl)anthranilate isomerase	–	–	–	–	–	–	–	–	–
tr|A0A1D6F1X1	Phospho-2-dehydro-3-deoxyheptonate aldolase	–	–	–	–	–	–	–	–	–

Note: SW-1 presents 12–5 × IE034

Therefore, it can be concluded that the impact of stacked-trait development via conventional breeding on the maize proteome was less significant than those of genetic modification or genotype. In addition, stacked-trait development via conventional breeding did not have a significant impact on the maize proteome.

## Discussion

A transcript level reduction of approximately 50% of each target foreign gene was observed in 12–5 × IE034 relative to the levels in the parents 12–5 and IE034. Similarly, Agapito-Tenfen et al. [23] observed significant reductions (from 29 to 41%) in the transcript levels of three transgenes in stacked varieties relative to the levels in parental single event varieties. Homologous ubiquitin promoters control foreign gene expression in the stacked line 12–5 × IE034. The observed reduction may be due to homology-dependent gene silencing resulting from the introduction of transgenes [25–30]. Gene silencing mediated by 35S promoter homology is a common problem and occurs in tagged lines from different collections [31]. Alternatively, the reduction in gene expression at the transcript level might be related to the high energetic demand of the cell. High energetic costs occur when foreign genes are driven by constitutive promoters in transgenic plants [32, 33]. Another possible reason may be that the foreign genes in 12–5 × IE034 are heterozygotes. Changes in the expression level may cause environmental or food safety concerns. Therefore, in the safety assessment, it is necessary to investigate the expression level of foreign genes in stacked lines.

Natural variation is widespread in the plant proteome. Nonspecific proteome profile and specific protein analyses have shown that protein expression is impacted by genotype and environmental factors [11, 34–37]. Lehesranta et al. [38] found that most detected proteins exhibited significant differences between one or more GM potato varieties and landraces. Agapito-Tenfen et al. [23] reported that protein changes in stacked transgenic maize differed significantly from those of single event lines and a conventional counterpart. In the present study, the proteomic profiles of different traditional maize varieties, stacked transgenic maize and its parents were analyzed using protein iTRAQ technology. A total of 102–380 differentially abundant proteins were detected among the five traditional maize varieties from different provinces. Seventy-two and 87 differentially abundant proteins were found between 12 and 5 × IE034 and its single-trait parents 12–5 and IE034, respectively. These proteins are primarily associated with the same KEGG pathway. In addition, no new non-target proteins were found in a comparison between 12 and 5 × IE034 and the traditional varieties. These results indicated that the impact of stacked-trait development via conventional breeding on the maize proteome was less significant than that of genetic manipulation or natural variation among maize varieties.

It is common to combine beneficial traits when breeding new crop varieties. Scientists combine multiple favorable traits, such as disease resistance, insect resistance and high yield, by leveraging hybrid vigor to develop new varieties that meet production demands. Gene introgression and gene pyramiding would inevitably occur in this breeding process. It has been suggested that over two decades, a total of 111 genes of 19 crops have been transferred from wild-type to cultivated species, of which 80% were related to disease resistance [39, 40]. The development of stacked-trait transgenic crops using single-trait transgenic crops as parents is substantially equivalent to conventional cross breeding which does not require molecular-level genetic manipulation. New varieties obtained through conventional cross breeding based on native crop varieties have a long, safe history as food/feed and are not required to undergo safety assessment prior to commercial cultivation. The process of stacked-trait development via conventional breeding is equivalent to the conventional cross breeding process except that transgenic crops are used as the parents. In addition, parent transgenic crops have undergone comprehensive safety assessments to ensure that they are as safe as recipient varieties. Therefore, it can be concluded that stacked-trait transgenic crops developed via conventional breeding do not present higher risks regarding food and feed safety than do their parents [10, 41].

Evaluations of substantial equivalence are essential for the commercial release of transgenic crops, and substantial equivalence is an important concept in transgenic crop safety assessments [42, 43]. Substantial equivalence has been widely used in safety assessments of transgenic crops worldwide [44]. The substantial equivalence concept also applies to stacked-trait transgenic crops developed via conventional breeding. Comparative analyses of 15 agronomic characteristics such as pollen viability, yield, stalk and root lodging, seedling vigor, disease resistance, insect resistance, and herbicide tolerance of stacked-trait transgenic MON 89034 × TC1507 × NK603 × DAS-40278–9 maize developed via conventional breeding indicated that transgenic maize was equivalent to conventional maize except in its targeted traits [45]. Geography- and season-related natural variation may change soybean components, and the impact of natural variation on soybean components such as isoflavones, fatty acids and vitamin E was shown to be more significant than that of genetic manipulation [46].

Thus, a breeding stack of two or more transgenic events is, in essence, a traditional breeding process. The stacking process does not involve gene transfer in vitro. In addition, the single-trait event varieties used as parents often undergo rigorous safety assessment before commercial release. Thus, the safety assessment of stacked GMCs should adopt simplified procedures based on the safety assessment of the single-trait parents. The producer should provide data related to genetic stability, foreign gene expression and the interaction between target genes as well as regulatory elements on a case-by-case basis.

## Conclusions

Stack breeding is substantially equivalent to the traditional breeding process. It does not affect on the insertion site, copy number or genetic stability of the foreign gene. Foreign gene expression in stacked transgenic maize is less significant than that in its parents. Proteomic profiling showed that the impact of stacked breeding on the maize proteome was less significant than that of genotypic differences. This is the first report on the comparative proteomic profiling of stacked versus different maize varieties. This result provides a theoretical basis for the development of a safety assessment approach to stacked-trait transgenic crops in China.

## Methods

### Plant materials

The transgenic insect-resistant maize IE034 with *cry1Ie* and nontransgenic maize Z31 (recipient of IE034) varieties were obtained from the Guoying Wang Lab of the Crop Science Institute, Chinese Academy of Agricultural Sciences (CAAS). The insect-resistant and herbicide-tolerant transgenic maize 12–5 with *cry1Ab/cry2Aj* and *G10-EPSPs* and Z58 (recipient of 12–5) varieties were obtained from the Zhicheng Shen Lab of Zhejiang University. The 12–5 × IE034 (SW-1) line was an F1 generation stacked from 12 to 5 and IE034. The plants containing all the foreign genes from 12 to 5 and IE034 were selected from 12 to 5 × IE034 and self-crossed to obtain 12–5 × IE034-F2 (SW-2). Therefore, 12–5 × IE034-F3 (SW-3) was a self-crossing offspring of 12–5 × IE034-F2 containing all the foreign genes. The nontransgenic maize varieties Sanmingzi ((SM, originated in Hebei Province, China), Daqingke (DQ, originated in Heilongjiang Province, China), and Baimaya (BM, originated in Guangdong Province, China) were provided by Prof Yunsu Shi Lab at the Crop Science Institute, CAAS.

### PCR detection of stacked-trait GM maize 12–5 × IE034

Genomic DNA (gDNA) was extracted from the leaves of transgenic maize using a broad-spectrum plant genomic DNA quick extraction kit (Tiangen Biotech (Beijing) Co., Ltd.) and was used to conduct multiple PCR detection of three target genes, *cry1Ab/cry2Aj*, *cry1Ie* and *G10-EPSPs*. The operating steps and primers used in this study were as previously described by Zhang et al. [47].

### Copy number determination of foreign genes in stacked-trait GM maize 12–5 × IE034

The following PCR primers and probes were designed based on the sequences of the *cry1Ab/cry2Aj* and *cry1Ie*: AB-F (GAGCCTGTTCCCCAACTACG), AB-R (GGTGTAGATGGTGATGCTGTTC), AB-probe (HEX-ACTACGACAGCCGCACCTACCCCAT-BHQ-1), IE-F (AACCCCGACAAGCACCAGAG), IE-R (GGAAGTCCTCGTGGTTGATGTT) and IE-probe (HEX-CACCAGAGCCTGAGCAGCAACGCC-BHQ-1). The following primers and probes were designed using the maize single-copy native gene *hmga* as a reference gene and by referring to the national standard GB/T1945.7–2004: HMG-F (TTGGACTAGAAATCTCGTGCTGA), HMG-R (GCTACATAGGGAGCCTTGTCCT) and HMG-probe (6-FAM-CAATCCACACAAACGCACGCGTA-BHQ-1). A 20-μL probe-digital PCR system (containing 10 μL of 2x ddPCR Super Mix for Probes, 1.4 μL of forward primer (700 nM), 1.4 μL of reverse primer (700 nM), 0.5 μL of probe (FAM/HEX) (250 nM), 1 μL of DNA (25 ng/μL), and 5.7 μL of H_2_O) was prepared. The well-mixed reaction system was added to a droplet generator to obtain micro-droplets, which were then transferred to a 96-well plate with a specific heat seal and incubated for 10 s at 180 °C. PCR was performed using a QX200 platform (Bio-Rad, USA) under the following conditions: pre-denaturation (94 °C, 10 min); denaturation (94 °C, 30 s), annealing and extension (62 °C, 60 s) cycles; and incubation (98 °C, 10 min). Upon completion of the reaction, a micro-droplet reader was used to read the signal. QuantaSoft software (Bio-Rad, USA) was used to analyze the test results. The T-DNA copy number was calculated using the following formula: copy number of T-DNA = T-DNA content/reference gene content [48].

### qRT-PCR detection of stacked-trait GM maize 12–5 × IE034

Maize total RNA was extracted using an EASYspin plant RNA extraction kit. The extracted RNA was reverse transcribed to cDNA through catalysis by reverse transcriptase. Using the first cDNA strand as a template, qRT-PCR was performed on an ABI7500 Real-Time System (Applied Biosystems, USA) using a SYBR Premix Ex Taq kit (TaKaRa, Dalian, China). For qRT-PCR analysis, the *actin* gene was used as an internal control, and the relative quantification method was used to assess the fold changes of the target genes. Five biological and three technical replicates were performed for each sample. The primers AC200F (ATGTTTCCTGGGATTGCCGAT) and AC200R (CCAGTTTCGTCATACTCTCCCTTG) were used for *actin* gene amplification. The primer pairs Ab-189-F (GAGCCTGTTCCCCAACTACG)/Ab-189-R (GGTGTAGATGGTGATGCTGTTC), GF1 (CCTCTGGCACCACTTTCGTGACCG)/GR1 (CGGAGCGTGGGACTTGATGTC), and IE256F (ATGTTTCCTGGGATTGCCGAT)/IE256R (CCAGTTTCGTCATACTCTCCCTTG) were used for *cry1Ab/cry2Aj*, *G10evo-EPSPs* and *cry1Ie* gene amplification, respectively. PCR was performed for 15 s at 95 °C, followed by 40 cycles of 95 °C for 5 s and 60 °C for 34 s.

Statistical analysis was carried out using SPSS software. Quantification cycle (Cq) average of RT-PCR for each biological replicate was calculated according to technical replicate results and used to perform statistical comparisons based on the standard deviation. The fold change data were log10 transformed because of their non-normal distribution. The fold change means obtained for different samples were compared using T-tests at *P* < 0.05 (SPSS software).

### Maize proteome analysis

Ten plants exhibiting normal growth and at the same stage (5–6 leaves) of each maize variety were selected for protein extraction. Soluble protein was extracted from the defined amount of maize leaves and then quantified using the Bradford method [49]. Proteins extracted from 10 plants of each variety were used to create protein pools (in equimolar ratios) before trypsin digestion. The mixture of protein solution was diluted to a final concentration of 1 μg/μL. Approximately 500 μL of 50 mM NH_4_HCO_3_ and 2 μL of trypsin were added to 100 μL of protein solution, and the mixture was incubated at 37 °C for 8–16 h. After trypsin digestion, the peptides were purified using a StrataX C18 column to remove the salt and dried in a freezing drier. The peptides were divided into six equal parts and labeled using an iTRAQ reagent 8-plex kit (Applied Biosystems, Waltham, US) according to the manufacturer’s instructions. Equal amounts of labeled samples were mixed and separated into 12 components using a Thermo DINOEX ultimate 3000 BioRS chromatograph and a Durashell C18 separation column (5 μm, 100 Å, 4.6 × 250 mm). An AB SCIEX nano LC-MS/MS (TripleTOF 5600 plus) mass spectrometer, AB SCIEX separation column (internal diameter: 75 μm, filling: 3 μm, ChromXP C18 column materials: 120 Å, length: 12 cm), NEW objective injection needle (internal diameter: 20 μm, tip diameter: 10 μm), and exigent ChromXP Trap Column (3 μm C18-CL, 120 Å, 350 μm × 0.5 mm) capturing column were used in the study.

Protein identification was performed using the ProteinPilot™ V4.5 software which is specific to the AB Sciex 5600 Plus system. The database used for the ProteinPilot™ V4.5 software analysis was the UniProt *Zea mays* database, which contains 142,200 proteins. The identified proteins that contained at least one unique peptide fragment and had a confidence level greater than 95% (unused score ≥ 1.3) were considered credible proteins. In addition, peptide fragments with a confidence level greater than or equal to 95% were deemed credible peptides.

iTRAQ quantification of the proteome was completed using ProteinPilot software. The ratio result output by ProteinPilot software is normalized by the median as the final difference multiple of protein. Proteins were considered significantly differentially expressed when they met both of the following conditions: i) the difference was 1.5 times or greater (up-regulated ≥1.5-fold or down-regulated ≤0.67-fold), and ii) the *P* value from the statistical significance test was less than or equal to 0.05 (The calculation of *P*-value is based on the ratio of peptide segments contained in identified proteins of each sample. it is an internal algorithm of the ProteinPilot software). Functional annotation of the significantly different proteins was completed by accessing the GO and KEGG databases.

## Data Availability

The datasets used and/or analyzed during this study are available from the corresponding author on reasonable request.

## Abbreviations

GMC: Genetically Modified Crops
GMO: Genetically Modified Organism
GO: Gene Ontology
iTRAQ: Isobaric tags for relative and absolute quantitation
KEGG: Kyoto Encyclopedia of Genes and Genomes
qRT-PCR: Quantitative Real-Time Polymerase Chain Reaction
RSD: Relative standard deviation
SPSS: Statistical Program for Social Science

## References

[1] ISAAA (2016). Global status of commercialized biotech/GM crops: 2016. ISAAA brief no. 52.

[2] Halpin C (2005). Gene stacking in transgenic plants--the challenge for 21st century plant biotechnology. Plant Biotechnol J.

[3] Paul L, Angevin F, Collonnier C, Messean A (2012). Impact of gene stacking on gene flow: the case of maize. Transgenic Res.

[4] Weber N, Halpin C, Hannah LC, Jez JM, Kough J, Parrott W (2012). Editor's choice: crop genome plasticity and its relevance to food and feed safety of genetically engineered breeding stacks. Plant Physiol.

[5] De Schrijver A, Devos Y, Van den Bulcke M, Cadot P, De Loose M, Reheul D (2007). Risk assessment of GM stacked events obtained from crosses between GM events. Trends Food Sci Technol.

[6] Kuiper HA, Kleter GA, Noteborn HP, Kok EJ (2001). Assessment of the food safety issues related to genetically modified foods. Plant J.

[7] Canadian Food Inspection Agency (2017). Regulating the environmental release of stacked plant products in Canada.

[8] Office of the Gene Technology Regulator of Australia (2011). Policy on licensing of plant GMOs in which different genetic modifications have been combined (or 'stacked') by conventional breeding.

[9] Food Safety Commission (2016). Regarding safty assessment of crossing of genetically modified plants.

[10] Steiner HY, Halpin C, Jez JM, Kough J, Parrott W, Underhill L (2013). Editor’s choice: evaluating the potential for adverse interactions within genetically engineered breeding stacks. Plant Physiol.

[11] Barros E, Lezar S, Anttonen MJ, van Dijk JP, Rohlig RM, Kok EJ (2010). Comparison of two GM maize varieties with a near-isogenic non-GM variety using transcriptomics, proteomics and metabolomics. Plant Biotechnol J.

[12] Coll A, Nadal A, Collado R, Capellades G, Kubista M, Messeguer J (2010). Natural variation explains most transcriptomic changes among maize plants of MON810 and comparable non-GM varieties subjected to two N-fertilization farming practices. Plant Mol Biol.

[13] Kogel KH, Voll LM, Schäfer P, Jansen C, Wu Y, Langen G (2010). Transcriptome and metabolome profiling of field-grown transgenic barley lack induced differences but show cultivar-specific variances. Proc Natl Acad Sci U S A.

[14] Montero M, Coll A, Nadal A, Messeguer J, Pla M (2011). Only half the transcriptomic differences between resistant genetically modified and conventional rice are associated with the transgene. Plant Biotechnol J.

[15] Liu Y, Zhang YX, Song SQ, Li J, Stewart CN, Wei W (2015). A proteomic analysis of seeds from Bt-transgenic *Brassica napus* and hybrids with wild *B. Juncea*. Sci Rep.

[16] Ren Y, Lv J, Wang H, Li L, Peng Y, Qu LJ (2009). A comparative proteomics approach to detect unintended effects in transgenic *Arabidopsis*. J Genet Genomics.

[17] Gong CY, Li Q, Yu HT, Wang Z, Wang T (2012). Proteomics insight into the biological safety of transgenic modification of rice as compared with conventional genetic breeding and spontaneous genotypic variation. J Proteome Res.

[18] Di Carli M, Villani ME, Renzone G, Nardi L, Pasquo A, Franconi R (2009). Leaf proteome analysis of transgenic plants expressing antiviral antibodies. J Proteome Res.

[19] Zolla L, Rinalducci S, Antonioli P, Righetti PG (2008). Proteomics as a complementary tool for identifying unintended side effects occurring in transgenic maize seeds as a result of genetic modifications. J Proteome Res.

[20] Sestili F, Paoletti F, Botticella E, Masci S, Saletti R, Muccilli V (2013). Comparative proteomic analysis of kernel proteins of two high amylose transgenic durum wheat lines obtained by biolistic and agrobacterium-mediated transformations. J Cereal Sci.

[21] Chen H, Bodulovic G, Hall PJ, Moore A, Higgins TJ, Djordjevic MA (2009). Unintended changes in protein expression revealed by proteomic analysis of seeds from transgenic pea expressing a bean alpha-amylase inhibitor gene. Proteomics.

[22] Rocco M, Corrado G, Arena S, D'Ambrosio C, Tortiglione C, Sellaroli S (2008). The expression of tomato prosystemin gene in tobacco plants highly affects host proteomic repertoire. J Proteome.

[23] Agapito-Tenfen SZ, Vilperte V, Benevenuto RF, Rover CM, Traavik TI, Nodari RO (2014). Effect of stacking insecticidal cry and herbicide tolerance epsps transgenes on transgenic maize proteome. BMC Plant Biol.

[24] Wang XJ, Zhang X, Yang JT, Wang ZX (2018). Effect on transcriptome and metabolome of stacked transgenic maize containing insecticidal cry and glyphosate tolerance epsps genes. Plant J.

[25] Fagard M, Vaucheret H (2000). (trans) gene silencing in plants: how many mechanisms?. Annu Rev Plant Biol.

[26] Park YD, Papp L, Moscone EA, Iglesias VA, Vaucheret H, Matzke AJM (1996). Gene silencing mediated by promoter homology occurs at the level of transcription and results in meiotically heritable alterations in methylation and gene activity. Plant J.

[27] Matzke MA, Matzke AJ (1998). Gene silencing in plants: relevance for genome evolution and the acquisition of genomic methylation patterns. Novartis Found Symp.

[28] Kohli A, Twyman RM, Abranches R, Wegel E, Stoger E, Christou P (2003). Transgene integration, organization and interaction in plants. Plant Mol Biol.

[29] Weld R, Heinemann J, Eady C (2001). Transient GFP expression in *Nicotiana plumbaginifolia* suspension cells: the role of gene silencing, cell death and T-DNA loss. Plant Mol Biol.

[30] Cogoni C, Macino G (1999). Homology-dependent gene silencing in plants and fungi: a number of variations on the same theme. Curr Opin Microbiol.

[31] Daxinger L, Hunter B, Sheikh M, Jauvion V, Gasciolli V, Vaucheret H (2008). Unexpected silencing effects from T-DNA tags in *Arabidopsis*. Trends Plant Sci.

[32] Rus AM, Estañ MT, Gisbert C, Garcia-Sogo B, Serrano R, Caro M (2001). Expressing the yeast HAL1 gene in tomato increases fruit yield and enhances K+/Na+ selectivity under salt stress. Plant Cell Environ.

[33] Grover A, Aggarwal PK, Kapoor A, Katiyar-Agarwal S, Agarwal M, Chandramouli A (2003). Addressing abiotic stresses in agriculture through transgenic technology. Curr Sci.

[34] Ruebelt MC, Lipp M, Reynolds TL, Schmuke JJ, Astwood JD, DellaPenna D (2006). Application of two-dimensional gel electrophoresis to interrogate alterations in the proteome of gentically modified crops. 3. Assessing unintended effects. J Agric Food Chem.

[35] McClain S, Stevenson SE, Brownie C, Herouet-Guicheney C, Herman RA, Ladics GS (2018). Variation in seed allergen content from three varieties of soybean cultivated in nine different locations in Iowa, Illinois, and Indiana. Front Plant Sci.

[36] Stevenson SE, Woods CA, Hong B, Kong X, Thelen JJ, Ladics GS (2012). Environmental effects on allergen levels in commercially grown non-genetically modified soybeans: assessing variation across North America. Front Plant Sci.

[37] Agapito-Tenfen SZ, Guerra MP, Wikmark OG, Nodari RO (2013). Comparative proteomic analysis of genetically modified maize grown under different agroecosystems conditions in Brazil. Proteome Sci.

[38] Lehesranta SJ, Davies HV, Shepherd LV, Nunan N, McNicol JW, Auriola S (2005). Comparison of tuber proteomes of potato varieties, landraces, and genetically modified lines. Plant Physiol.

[39] Hajjar R, Hodgkin T (2007). The use of wild relatives in crop improvement: a survey of developments over the last 20 years. Euphytica.

[40] Fernie AR, Tadmor Y, Zamir D (2006). Natural genetic variation for improving crop quality. Curr Opin Plant Biol.

[41] Parrott W, Chassy B, Ligon J, Meyer L, Petrick J, Zhou J (2010). Application of food and feed safety assessment principles to evaluate transgenic approaches to gene modulation in crops. Food Chem Toxicol.

[42] Codex Alimentarius Commission (2003). Guideline for the conduct of food safety assessment of foods derived from recombinant-DNA plants.

[43] Organisation for Economic Co-operation and Development (OECD) (1993). Safety evaluation of foods derived by modern biotechnology: concepts and principles.

[44] Kuiper HA, Kleter GA, Noteborn HP, Kok EJ (2002). Substantial equivalence--an appropriate paradigm for the safety assessment of genetically modified foods?. Toxicology.

[45] de Cerqueira DTR, Schafer AC, Fast BJ, Herman RA (2017). Agronomic performance of insect-protected and herbicide-tolerant MON 89034 x TC1507 x NK603 x DAS-40278-9 corn is equivalent to that of conventional corn. GM Crops Food.

[46] Berman KH, Harrigan GG, Nemeth MA, Oliveira WS, Berger GU, Tagliaferro FS (2011). Compositional equivalence of insect-protected glyphosate-tolerant soybean MON 87701 x MON 89788 to conventional soybean extends across different world regions and multiple growing seasons. J Agric Food Chem.

[47] Zhang X, Dong YF, Wang XJ, Wang ZX (2017). Target trait test and multiple PCR detection of insect resistance and herbicide tolerance transgenic maize SW-1. Current Biotechn.

[48] Weng H, Pan A, Yang L, Zhang C, Liu Z, Zhang D (2004). Estimating number of transgene copies in transgenic rapeseed by real-time PCR assay with HMG I/Y as an endogenous reference gene. Plant Mol Biol Report.

[49] Bradford MM (1976). A rapid and sensitive method for the quantitation of microgram quantities of protein utilizing the principle of protein-dye binding. Anal Biochem.

